# Time to recovery and its predictors among children 6–59 months with acute malnutrition admitted to community inpatient therapeutic feeding centers in Katsina State, Northwest Nigeria: a retrospective review of health records (2010–2016)

**DOI:** 10.1186/s41043-023-00352-y

**Published:** 2023-02-17

**Authors:** Friday Ilop Joseph, Adewale Falade, Jane Earland

**Affiliations:** 1 Department of Paediatrics, Federal Teaching Hospital, Katsina, Katsina State Nigeria; 2 Department of Public Health, Obafemi Awolowo University, Ile-Ife, Osun State Nigeria; 3 Department of Public Health and Policy, School of Medicine, Faculty of Health and Life Sciences, University of Liverpool, Liverpool, UK

**Keywords:** Complicated SAM, Under-five, Recovery time, CMAM, Nigeria

## Abstract

**Background:**

Severe acute malnutrition (SAM) among children under five years of age remains a huge public health and economic burden in Sub-Saharan Africa. We investigated time to recovery and its predictors among children aged 6 to 59 months admitted into Community-based Management of Acute Malnutrition (CMAM) stabilisation centres for complicated severe acute malnutrition and whether the outcomes met the minimum Sphere standards.

**Methods:**

The study was a retrospective cross sectional quantitative review of data recorded in six CMAM stabilization centres registers in four Local Government Areas, Katsina state, Nigeria from September 2010 to November 2016. Records of 6925 children, aged 6–59 months with complicated SAM were reviewed. Descriptive analysis was used to compare performance indicators with Sphere project reference standards. Cox proportional hazard regression analysis was used to estimate the predictors of recovery rate at *p* < 0.05 and Kaplan–Meier curve to predict the probability of surviving different forms of SAM.

**Results:**

Marasmus was the most common form of severe acute malnutrition (86%). Overall, the outcomes met the minimum sphere standards for inpatient management of SAM. Children with oedematous SAM (13.9%) had the lowest survival rate on Kaplan–Meier graph. The mortality rate was significantly higher during the ‘lean season’—May to August (Adjusted Hazard Ratio (AHR) = 0.491, 95% CI = 0.288–0.838). MUAC at Exit (AHR = 0.521, 95% CI = 0.306–0.890), marasmus (AHR = 2.144, 95% CI = 1.079–4.260), transfers from OTP (AHR = 1.105, 95% CI = 0.558–2.190) and average weight gain (AHR = 0.239, 95% CI = 0.169–0.340) were found to be significant predictors of time-to-recovery with p values < 0.05.

**Conclusion:**

The study showed that, despite a high turnover of complicated SAM cases in the stabilization centres, the community approach to inpatient management of acute malnutrition enabled early detection and reduced delays in access to care of complicated SAM cases. In the face of health workforce shortage in rural communities to provide pediatric specialist care for SAM children, we recommend task shifting to community health care workers through in service training could bridge the gap and save more lives of children dying from the complication of SAM in rural communities in Nigeria.

## Introduction

Globally, about 150 million under five children are estimated to be too short for their age or stunted and about 50 million too thin for their height or wasted [1]. Most of the world’s wasted children are living in South Asia and Sub-Saharan Africa [1, 2] and account for about 50% of direct or indirect cause of global under-five mortality [3, 4].

Malnutrition, in all its forms, is due to deficiency, excesses or imbalances in a person’s intake of energy and/ or nutrients. Malnutrition includes under nutrition, micronutrient-related malnutrition and overweight, obesity and diet-related non-communicable diseases [5, 6]. Undernutrition includes wasting (acute malnutrition), stunting (chronic malnutrition) and underweight (both acute and chronic malnutrition) and micronutrient deficiency. Acute malnutrition can present as wasting (marasmus), oedema (kwashiorkor), or both wasting and oedema (marasmic kwashiorkor) [7, 8].

Adequate nutrition is crucial for a child’s physical and cognitive development, especially in the first 1,000 days of live [9, 10]. The causes of undernutrition are complex and interwoven. Immediate causes include lack of access to highly nutritious foods, especially in a low socio-economic context and infectious diseases, including pneumonia, measles and malaria. Other causes include poor maternal health and nutrition, and/or inappropriate infant and young child feeding and care in the early life (WHO, 2018). The World Health Organization (WHO) defines Severe Acute Malnutrition (SAM) in children aged 6–59 months as weight for height/length  < − 3SD, the presence of any bilateral pitting oedema (when all other non-nutritional causes are excluded), and/or a mid-upper arm circumference (MUAC) < 11.5 cm.

Before the advent of CMAM in 2007, therapeutic feeding interventions depend on the traditional hospital-based Therapeutic Feeding Centers (TFCs) as the primary mode of interventions [11]. SAM patients are admitted for a period of three weeks or longer and caregivers are required to stay with their malnourished children for that period in the centres. The limitations of the hospital-based approach include delay in accessing optimal care for a condition affecting large numbers of children, particularly when hospital capacity is poor [12–14]. Moreover, hospital stays of several weeks for a child and mother are disruptive for families, especially when the mother’s is essential for the economic survival of the household. In a hospital setting where SAM patient are managed with other children with infectious disease, prolonged hospital stay expose them to nosocomial infection [11–13]

Community Management Acute Malnutrition (CMAM) is designed to address the limitations of hospital-based inpatient care [13]. CMAM intervention use decentralized networks of outpatient treatment sites (located at existing primary health-care facilities) for SAM without medical complications, small inpatient stabilization centres (SCs) for SAM with medical complications, and large numbers of community-based volunteers to provide case detection and follow-up of patients in their home environments [15]. Medical complications include poor appetite, signs of infection, severe oedema, hypothermia, lethargy, hypoglycaemia, vomiting, diarrhoea with dehydration, severe anaemia [7, 16]. The minimum international standard set to evaluate the quality of management of SAM according to Sphere is a cure rate >75% and death rate <10% [16].

Northern Nigeria has been experiencing drought and chronic food insecurity for the past three decades. The situation in the last decades has worsened due to insurgency and Herder/farmer crises [17]. In the most recent survey conducted in Nigeria [18], 37% of children were found to be stunted, 7% were wasted and 22% were overweight. Only 29% of children under the age 6 months were breastfed exclusively [18, 19]. In the 2018 NDHS, there were marked variations by zone in the prevalence of stunting and wasting. The North West region of Nigeria, where this study was conducted, had the highest proportions of stunted (57%) and wasted children (9%) while the South East had the least, with 18% of children found to be stunted and 5% wasted [18].

From 2010 to 2016, Save the Children implemented an integrated CMAM programme for under–five children in Baure, Daura, Dutsi and Zango Local Government Areas (LGA), in Katsina state, Northwest region of Nigeria. The success of a CMAM programme relies on early case finding combined with effective follow up actions at the community level. Community volunteers were selected and trained to identify malnourished children using a Mid Upper Arm Circumference (MUAC) strip and refer children to the Outpatient Therapeutic Programme (OTP), depending on the severity of malnutrition [7, 20]. In the OTP, SAM cases that developed medical complications were referred to the SC for inpatient care [7, 16]. At the health facility level, health care workers were trained annually in the WHO treatment guidelines for the management of SAM with comorbidity in the in-patient setting [4].

So, the objectives of this study were to investigate the time to recovery and it’s predictors among under-five children admitted into SCs and managed for complicated SAM; and also to investigate whether the outcomes met the minimum Sphere standards and compare with intervention where tertiary health facilities were used for inpatient management of complicated SAM.

## Methods

### Study design and period

The study was a retrospective cross sectional quantitative review of data recorded in CMAM inpatient centres registers between September 2010 and November 2016.

### Study setting

This study was undertaken in two secondary healthcare facilities (General Hospital Daura and General Hospital Baure) and four primary healthcare facilities (Comprehensive Health Centre Daura, Comprehensive Health Centre Dutsi, Comprehensive Health Centre Zango, Maternal and Child Health Centre Zango) designated as SCs across four Local Government Area (LGA), namely Baure, Daura, Dutsi and Zango in Katsina state, Northwest region of Nigeria. The stabilisation centres are situated in rural communities and admit only under five children with complicated SAM. The communities served by the health facilities are predominantly Hausa/Fulani-Muslims living in rural areas. The majority are agrarian and nomad. The total bed capacity in the six SCs was 60. In the four primary healthcare facilities, the wards were managed by Community Health Workers (CHWs) trained in the inpatient management of SAM, while the two secondary health facilities were managed by nurses. These health workers received yearly refresher training in the inpatient management of SAM.

All the children were managed using the WHO 10 step to inpatient management of SAM. For children 6–59 months, once the child regain appetite and were clinically stable, they were transferred to the OTP closest to the child’s home, to continue nutritional rehabilitation with peanut based ready-to-use food until full recovery. HIV serology for children older than 19 months of age was measured using a rapid diagnostic test. However, the majority of the participants were not screened because of the inconsistency in the supplied of the kits. All children found to be HIV infected were referred to a secondary health facility for antiretroviral therapy (ART).

### Population and sampling procedures

**Fig. 1:**
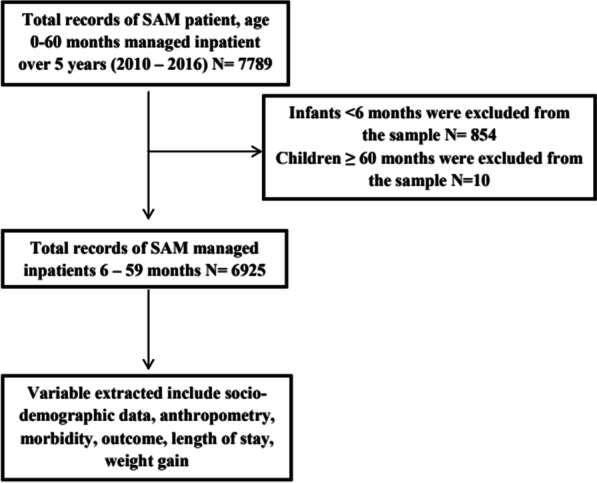
Flow chart showing sampling procedure

All children aged 6–59 months admitted to the SCs between 2010 and 2016 were eligible for the study. Admission to SCs is based on the presence of bilateral pitting oedema and /or MUAC < 11.5 cm for children 6–59 months with medical complications. The initial sample size was 7789 children age zero to 60 months on the CMAM registers. However, the de facto eligible sample was 6925 due to excluding records with incomplete information like weight gain, length of stay and HIV status. Children were either admitted directly from the community or transferred from OTP centres. The sampling procedure is shown in Fig. 1.

### Inclusion and exclusion criteria

All Children in the register were eligible for the study while excluding infants less than 6 months and children age greater than 59 months.

### Data collection

The data were extracted from the CMAM inpatient records directly into Microsoft Excel for cleaning and then exported to the Statistical Package for Social Science (SPSS) version 23 for analysis.

### Definitions

Severe Acute Malnutrition: Mid upper Arm circumference < 11.5 cm and/or oedema (excluding non-nutritional cause of oedema).

Outpatient therapeutic programme: A component of CMAM that care for uncomplicated SAM.

Inpatient therapeutic programme: A component of CMAM that care for complicated SAM in stabilization centres.

Length of stay: The number of days a patient stays in the health facility before achieving an outcome.

Outcome: Stabilized/recovered, death or defaulted.

Recovery: SAM patient that has regained a good appetite and evidence of medical complications being resolved.

Defaulter: SAM patient that left treatment before recovery.

Death: SAM patient who died during the course of inpatient therapy.

### Study variables

Dependent variables: outcome variables were stabilised/recovered; death and defaulter, length of stay (days) and average weight gain (g/kg/day).

Independent variables: the independent variables were socio-demographic data (age, gender, and place of admission), anthropometry at admission and discharge (MUAC, weight), forms of malnutrition (marasmus, marasmus-kwashiorkor and kwashiorkor), facility of admission, and month of admission.

### Data analysis

The Kolmogrov Smirnov test of normality was used to check the normality of the distributions for the continuous variables which indicated that the variables were normally distributed The Multicollinearity test between independent variables indicated that there was no correlation between variables based on a variance inflation factor of less than 10. Data were described using frequency distribution and measures of central tendency and dispersion. Kaplan–Maier Curve and Long rank tests were used to estimate cumulative survival probability and to compare survival status probability across different groups.

Bivariable and multivariable Cox regression were used to identify predictors of time-to-recovery and outcome. Variables with a *p* value less than 0.05 during bivariate analysis were included in the multivariate analysis. A sensitivity analysis was done to ascertain whether the findings of this study would have been different if the missing data for weight at exit and type of SAM (Table 1) had been included in the analysis. A dichotomous variable was then created using the total number of missing and present data in the dependent variable mortality. The analysis shows that generally the known data was more than the missing. Consequently, it can be inferred that the results would not have changed significantly if all the missing data were added to the analysis.

**Table 1:** Bivariable Cox proportional hazard regression model for predictors of death from complicated SAM in Northwest Nigeria

Variable	SAM			
	All SAM (6925) *n*%	Alive (6925) *n*%	Death (285) *n*%	Crude Hazard Rate (95% CI)
Sex (*n* = 6925)				
Male	3,570 (52%)	3435 (96%)	135 (4%)	1
Female	3,352 (48%)	3202 (96%)	150 (4%)	0.883 (0.691–1.130)
Age(month) (*N* = 6925)				
6–24	6.101 (88%)	5850 (96%)	251 (4%)	1
25–59	824 (12%)	790 (96%)	31 (4%)	0.985 (0.683–1.421)
Month of Admission (*N* = 6925)				
January–April	1,410 (20%)	1375 (98%)	35 (2%)	1
May–August	2,735 (42%)	2600 (95%)	135 (5%)	0.542(0.363–0.809)*
September–December	2,772 (38%)	2657 (96%)	115 (4%)	1.228(0.942–1.599)*
Weight (kg) at Admission (*N* = 6924)				
< 6	4,067 (59%)	3904 (96%)	163 (4%)	1
6 +	2,857 (41%)	2735 (96%)	122 (4%)	1.136 (0.887–1.457)
Weight (kg) at Exit (*N* = 6621)				
< 6	3343 (50%)	3203 (96%)	140 (4%)	1
6 +	3278 (50%)	3170 (97%)	108 (3%)	0.850 (0.652–1.108)
MUAC (cm) at Admission (*N* = 6925)				
< 11.5	6102 (88%)	5837 (96%)	265 (4%)	1
11.5 +	823 (12%)	803 (97%)	20 (3%)	1.731 (1.072–2.796)*
MUAC (cm)at Exit (*N* = 6925)				
< 11.5	5376 (78%)	5114 (95%)	262 (5%)	1
11.5 +	1549 (22%)	1526 (98%)	23 (2%)	3.057 (1.974–4.734)*
Type of SAM (*N* = 6846)				
Marasmus	1639 (24%)	1517 (93%)	122 (7%)	0.417 (0.325–0.536)*
				1
Marasmic-kwashiorkor	41 (1%)	39 (95%)	2 (5%)	2.305 (.318–16.703)
				1
Kwashiorkor	223 (3%)	208 (93%)	15 (7%)	0.642(0.359 -.1.148)
				1
Children transferred from OTP	4943 (72%)	4801 (97%)	142 (3%)	2.377(1.855–3.047)*
				1
Residence (*N* = 6925)				
Baure^+^	806 (12%)	774 (96%)	32 (4%)	1
Dutsi*	875 (13%)	841 (96%)	34 (4%)	1.543 (1.417–1.680)*
Daura^+*^	2394 (35%)	2319 (97%)	75 (3%)	1.504 (1.361–1.663)*
Zango*	2850 (41%)	2706 (95%)	144 (5%)	1.247 (1.147–1.356)
Weight gain (g/kg/day) (N = 5866)				
< 8	2570 (44%)	2425 (94%)	175 (6%)	1
8 +	3296 (56%)	3252 (99%)	44 (1%)	0.210 (0.149–0.295)*

Owing to missing data, values may not add up

*P value < 0.05

## Ethical consideration

Institutional Review Board (IRB) approval was obtained from Katsina State Health Research Ethics committee/Operations Research Advisory Council (ORAC) upon submission of the study design and data collection instruments prior to the commencement of the survey. The ORAC is domiciled in the Katsina State Ministry of Health. As this was conducted on anonymized secondary data, patient informed consent was waived. To ensure their confidentiality, study participants were represented by codes.

## Results

### Population and demographic characteristics, anthropometry and admission outcomes

**Fig. 2:**
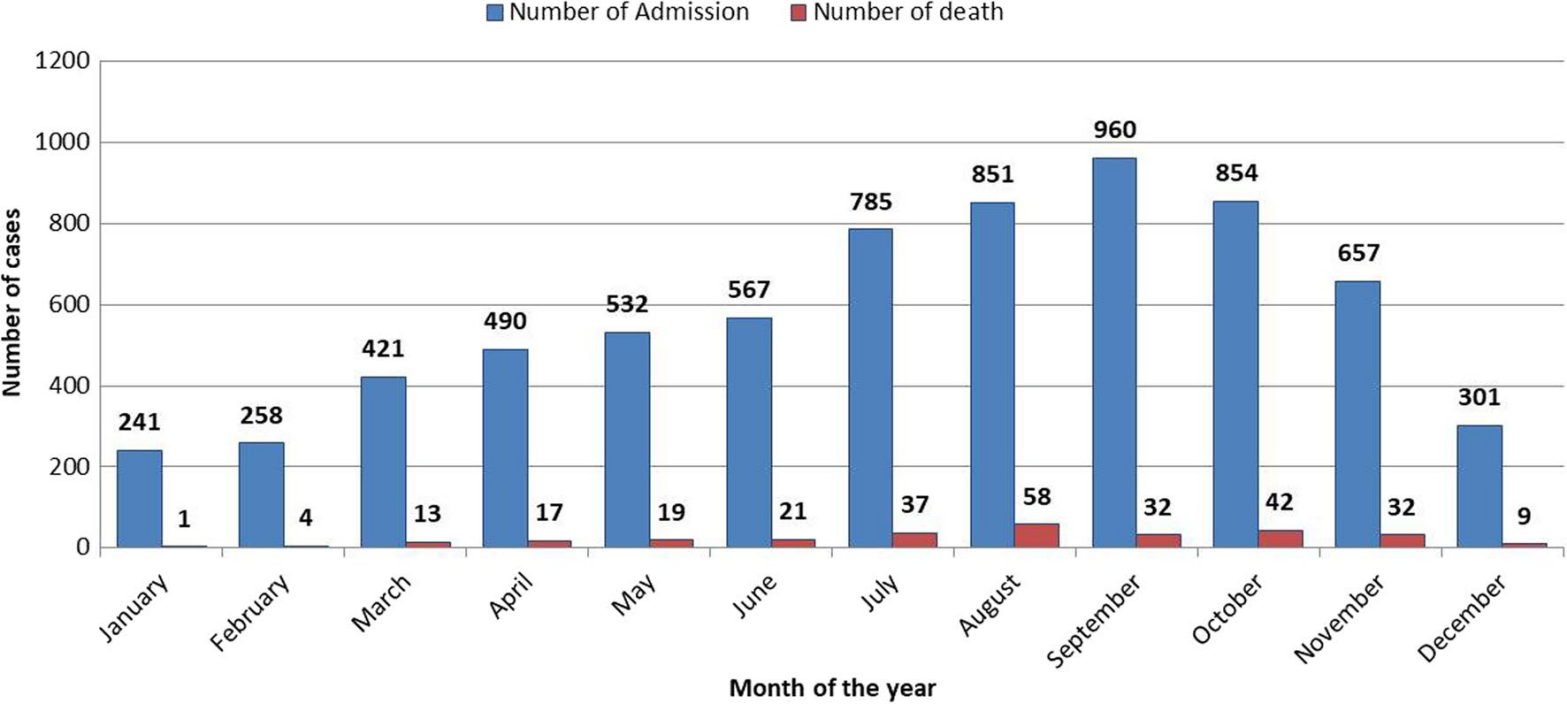
Distribution of the number of admission and death of complicated SAM cases per month

The number of under–five children enrolled into the study were 6925; the males constituted 52% (3570), with a boys-to-girls ratio of 1.06. The median age of the sample was 16 months (IQR 10–24 months). The mean (± SD) age of the children was 17.58 months (± 9.75) and 88% were ≤ 24 month. Transfers from OTP were 65% (4943) while 35% were referrals from the communities. Marasmus constituted 86.0% of direct admissions, followed by kwashiorkor (11.8%) and marasmus kwashiorkor (2.1%). The median MUAC at admission was 10.17 cm (IQR 9.50–11.00), with 66.5% of the sample having a MUAC of less than 11.00 cm. The mode was 11.0 cm. The mean weight at admission was 5.67 kg (± 1.88 SD), median weight was 5.60 kg (IQR 4.70–6.50) and mode 6.00 kg. Most admissions (42%) occurred within the ‘lean season’ (May to August) with corresponding higher mortality figures than in other months (Table 1 and Fig. 2).

### Changes to MUAC and weight during admission

To test the hypothesis that there was no difference between the means of the MUAC (M = 10.19 cm, SD = 1.30) and weight at admission (M = 5.66, SD = 1.88), and MUAC (M = 10.526 cm, SD = 1.30) and weight at exit (M = 5.95, SD 1.60), a dependent sample t-test was performed. The correlations between the means of the MUACs and weights before and after intervention were estimated at r = 0.55, *p* < 0.001 and r = 54, *p* < 0.001 respectively, suggesting that the dependent sample t-test is appropriate in this case. The null hypothesis that the CMAM intervention did not cause any change in the mean of the MUAC and weight before and after was rejected, t (21.652) = 6279, *p* < 0.001 and t (13.718) = 6571, *p* < 0.001. Thus, the mean MUAC and weight at exit were statistically significantly higher than at admission.

### Treatment outcome

The records indicated that 5944 (86%) of SAM children aged 6–59 months were stabilised and transferred to OTP, which was above the minimum recovery rate of 75% recommended in the Sphere standards, while 285 (4%) died during treatment which was lower than the Sphere standards mortality rate <10%.

The mean length of stay in the SCs was 6.64 days (± 4.65), mode 4.00 and median length of stay for children who were discharged was 6.00 days (IQR 4.00 to 8.00 days). The majority of patients (69%) spent at least seven days as an inpatient. The average weight gain during the inpatient treatment phase was 6.8 g/kg/day for non-oedematous malnutrition (Table 2).

**Table 2:** Comparison of treatment outcome with SPHERE standard indictors

Discharge outcome	Result	SPHERE Standards
Transfer to OTP	86%	> 75%
Death	4%	< 10%
Defaulter	4%	< 15%
Average Length of stay	6.64 days	< 30 days
Weight gain (g/kg/day)	6.8 g/kg/day	≥ 8 g/kg/day

### Survival analysis

**Fig. 3:**
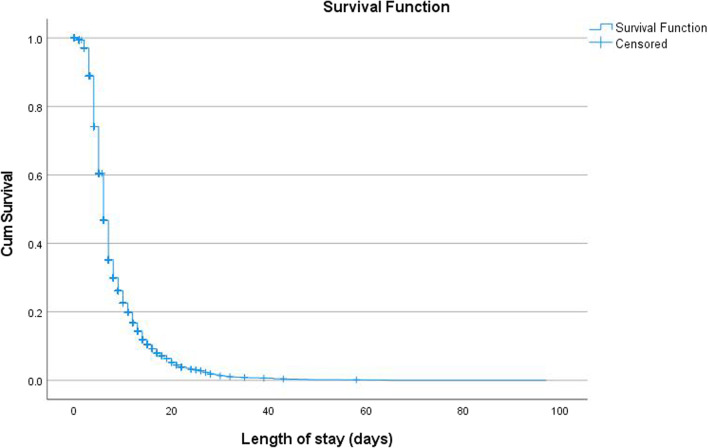
Survival curve for complicated SAM cases admitted into the Stabilisation Centres

**Fig. 4:**
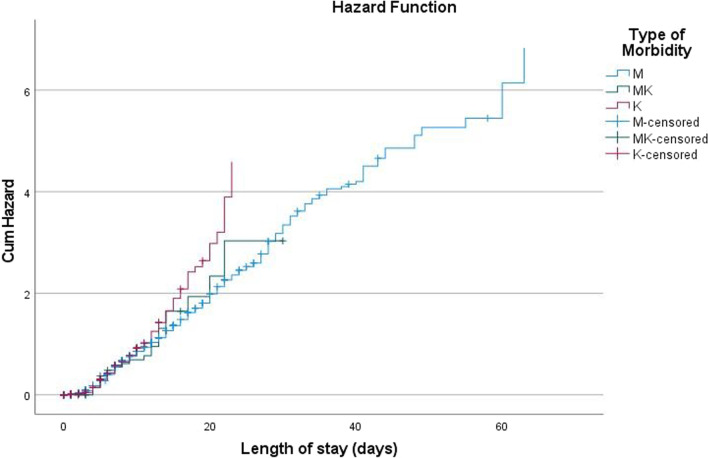
Kaplan-Meier failure estimates for type of acute malnutrition

There were 6925 children with SAM considered for survival analysis with a median recovery time of the entire cohort to be six days (95% CI 5.944–6.056). The greatest number of deaths (196) occurred within the first seven days of being admitted to the SCs. The cumulative probability of recovery at the end of one week was 95%; and recovery at the end of two week was 92%; surviving at the end of four weeks was 91% (Table 3 and Fig. 3). Kaplan–Meier failure curves showed that children with kwashiorkor had an increased risk of dying which was independent of other factors as shown in Fig. 4.

**Table 3:** Life table analysis of severely acutely malnourished children treated at CMAM stabilization centres Northwest Nigeria from September 2010 to November 2016

Interval start time (days)	Number entering interval	Number withdrawing during interval	Number exposed to risk	Death	Proportion dying	Proportion surviving	Cumulative proportion surviving at end of interval
0–7	5738	3328	4074.000	196	0.05	0.95	0.95
8–14	2214	1621	1403.500	45	0.03	0.97	0.92
15–21	548	288	404.000	7	0.02	0.98	0.91
22–28	253	51	227.500	0	0.00	1.00	0.91
29–35	202	34	185.000	3	0.02	0.98	0.89
36–42	165	40	145.000	0	0.00	1.00	0.89
43–49	125	7	121.500	0	0.00	1.00	0.89
50–56	118	4	116.000	0	0.00	1.00	0.89
57–63	114	8	110.000	0	0.00	1.00	0.89
64–70	106	16	98.000	1	0.01	0.99	0.88
71–77	89	87	45.500	2	0.04	0.96	0.84

### Identification of independent predictors of recovery time

Bivariate Cox regression analysis was run for the following independent variables: sex, age, month of admission, MUAC and weight at admission and discharge, type of malnutrition, weight gain and length of stay in the hospital. Subsequently, the bivariable analysis finding showed that the ‘lean season’—May to August (Crude Hazard Ratio (CHR) = 1.228, 95% CI = 0.942–1.599), MUAC at admission (CHR = 1.731, 95% CI = 1.072–2.796), MUAC at exit (CHR = 3.057, 95% CI = 3.057 (1.974–4.734), type of acute malnutrition—marasmus (CHR = 0.417, 95% CI = 0.325–0.536), transfer from OTP (CHR = 2.377, 95% CI 1.855–3.047) and change in weight (CHR = 0.210, 95% CI = 0.149–0.295) were found to be significant predictors of time-to-recovery with a *p* value < 0.05. Hence these variables were considered eligible for the multivariable Cox regression analysis. The month of admission (Adjusted Hazard Ratio (AHR) = 0.491, 95% CI = 0.288–0.838), MUAC at admission and exit(AHR = 0.521, 95% CI = 0.306–0.890), type of morbidity- marasmus (AHR = 2.144, 95% CI = 1.079–4.260), transfers from OTP(AHR = 1.105, 95% CI = 0.558–2.190), weight gain (g/kg/day) (AHR = 0.239, 95% CI = 0.169–0.340) were found to be independent predictors of recovery time in severely malnourished children admitted to the SCs (*p* < 0.05) as shown in Table 4.

**Table 4:** Multivariable cox regression of predictor of recovery in CMAM stabilization centres in Katsina State

Variable	Adjusted hazard ratio	*p* value
Month of admission		
January–April	1	0.009
May–August	0.491 (0.288–0.838)	0.009
September–December	1.118 (0.805–1.551)	0.506
MUAC (cm) at admission		
< 11.5	1	
11.5 +	0.990 (0.547–1.790)	0.974
MUAC (cm) at exit		
< 11.5	1	
11.5 +	0.521 (0.306–0.890)	0.017
Weight gain (g/kg/day)		
< 8	1	
8 +	0.239 (0.169–0.340)	< 0.001
Marasmus		
No	1	
Yes	2.144 (1.079–4.260)	0.029
Admitted from OTP		
No	1	
Yes	1.105 (0.558–2.190)	0.775

There were statistically significant differences in the survival rates among children admitted during the ‘lean season’ (May to August), MUAC at exit, marasmus and weight gain. Children with higher MUAC values at discharge and weight gain and with marasmus (rather than kwashiorkor or marasmic kwashiorkor) were more likely to survive before exiting the SCs.

## Discussion

The study revealed that the recovery from complicated SAM was 95.7% and 4.3% of children died and thus the minimum Sphere standards were met in the inpatient CMAM intervention within the study period. The low mortality in the study could be attributed to the community linkages with early detection of complications and prompt referral to the SCs. Previous  studies in Ethiopia [21, 22], Malawi [23] and Zambia [24] done in  tertiary hospitals, mortality rates were was 12.2%, 10.1% and 46%, respectively. In contrast to these previous studies, this study was undertaken in four primary health facilities managed by community health workers, and two secondary health facilities managed by nurses. These health workers received annual training in the inpatient management of acute malnutrition.

This study highlighted the importance of a community approach to the treatment of SAM in reducing deaths due to complicated SAM. The majority of undernourished children in Nigeria live in rural areas; early detection, timely referral and access to healthcare are key determinants to survival of complicated SAM. Tertiary health facilities have the expertise for child health care and treatment of complicated SAM. However, most tertiary health facilities are several kilometres away from these rural communities. Furthermore, difficult terrain with poor roads and dysfunctional referral systems are physical barriers to accessing tertiary health facilities in most developing countries [13, 14]. This may explain the reason for the high mortality in the reviewed studies. Drawing from the findings in this study, training community health workers and nurses in the inpatient management of complicated SAM and integrated management of childhood illness could save millions of under-five malnourished children in rural communities.

In this study the average length of stay was 7.42 days, which was far less than other studies in Ethiopia [25, 26], Ghana [27], India [28],Uganda [29], South Africa [30] and Zambia [31]. These could be attributed to the fact that inpatient centres are stabilization centres, and the stabilization phase of inpatient management is seven days, though the duration  varies depending on the severity of the complications[32]. Where there are  OTP centres  close to the child's homes, the rehabilitation phase can be completed in these centres. However, children may stay for a longer period in the SCs to continue rehabilitation phase with F100, an energy dense therapeutic milk where there are no OTP centres. The communities where this study was conducted have over 50 outpatient posts across these communities supported by Save the Children international. This is important in settings of high SAM caseloads like the one in our study. Higher length of stays in other studies may have been due to lack of outpatient therapeutic centres. However, there are risks of nosocomial infections and increased wait times in setting with a high number of complicated SAM cases.

The study revealed that the average weight gain of complicated SAM children in the stabilization centres was 6.8 g/kg body weight /day. This is below the Sphere standard minimum average weight gain ≥ 8 g/kg body weight/day and in similar studies conducted in Ethiopia (11.2 g/kg/day) and India (12.1 g/kg/day). The result may be attributed to the fact that the majority of SAM cases (69%) were discharged within the first seven days (Stabilisation phase) of inpatient care to continue rehabilitation phase in the OTP centres with energy dense plumpy nut. The stabilization phase includes a cautious approach to feeding, with F75, a special formula designed to help the child recover normal metabolic function and nutrition-electrolyte balance with minimal weight gain.

Marasmus was the most common type of acute malnutrition seen, similar to other studies in Ethiopia and Malawi [21–23]. However, some studies in Zambia and Sudan reported kwashiorkor to be the most common type of  acute malnutrition [24, 33]. Munthali et al. (2015) argued that one reason for this could be that the food in Zambia is carbohydrate based and that protein rich foods may not be available in the required quantities. However, the composition of the diet in rural Zambia is similar to that of Northwest Nigeria; therefore other reasons than diet may explain the  regional variation in the forms of acute malnutrition, as noted by several experts [6, 34]. This study also reviewed that SAM children with oedematous malnutrition have greater risk of mortality compare to non-oedematous. The majority of the children with Kwashiorkor died within seven days of admission. This finding is consistent with another study in Malawi [23] and highlight oedematous SAM as high risk form of Acute malnutrition.

The setting of the research is a rural area largely representative of the Sahel region with endemic malnutrition and seasonal peaks of acute malnutrition during the lean season (May to August) when food from the previous harvest are exhausted and there are  increased transmission of infectious diseases. The rainy season is a breeding period for malaria and high transmission rate for diarrhoea diseases among under five children. In this study, more admissions were in the lean season and malaria season, highlighting the role of inadequate food supply [35] and infections such as malaria [36–38] in increasing the morbidity and mortality of complicated SAM. In a study on the prevalence of malaria in the same region, malaria prevalence was highest in the ‘lean season’ with malaria cases over 50% higher than the yearly average [39, 40].

In previous studies the association between severity of clinical symptoms and SAM outcome in inpatient settings has been investigated [25, 26, 28, 30, 31, 41–44]. While the majority of studies were with patients admitted in tertiary hospitals with the technology and facilities to make definitive diagnosis of co morbidity, the SAM children in this study were managed in primary health care facilities designated as stabilization centres.

### Limitations of the study

This study provides overall burden and outcome among SAM patient managed as inpatients. However, there was no follow up of what happened once they are discharge from the SCs to the community. This is an opportunity for a cohort study to explore outcome of children managed in CMAM interventions. The retrospective nature of the data source with limited information recorded on the medical complications meant that the researchers could not identify the major causes of death among these children.

## Conclusion

The study demonstrates that the SCs for CMAM intervention in the rural communities of Katsina state met the minimum Sphere standards for inpatient care for complicated SAM cases except for average weight gain. The study highlighted MUAC at discharge, marasmus, ‘lean season’ (May to August) and change in weight were predictors of outcome for complicated SAM. The study revealed that community health workers play a vital role in ensuring access and care for complicated SAM, when they are appropriately trained and utilised in primary health facilities in rural communities. The findings also demonstrate the need for integrated management of childhood illnesses policy and intervention that factors the role of ‘lean season’ and seasonal malaria on the morbidity and mortality of severely malnourished under five children in this region.

## Data Availability

The datasets used and/or analyzed during the current study are available from the corresponding author on reasonable request.
